# Negative regulation of glial Tim‐3 inhibits the secretion of inflammatory factors and modulates microglia to antiinflammatory phenotype after experimental intracerebral hemorrhage in rats

**DOI:** 10.1111/cns.13100

**Published:** 2019-01-24

**Authors:** Zhou‐Qing Chen, Hao Yu, Hai‐Ying Li, Hai‐Tao Shen, Xiang Li, Ju‐Yi Zhang, Zhu‐Wei Zhang, Zhong Wang, Gang Chen

**Affiliations:** ^1^ Department of Neurosurgery & Brain and Nerve Research Laboratory The First Affiliated Hospital of Soochow University Suzhou China; ^2^ Department of Neurosurgery Nantong No.1 People Hospital Nantong China

**Keywords:** HIF‐1α, inflammation, intracerebral hemorrhage, microglia, Tim‐3

## Abstract

**Aims:**

To investigate the critical role of Tim‐3 in the polarization of microglia in intracerebral hemorrhage (ICH)‐induced secondary brain injury (SBI).

**Methods:**

An in vivo ICH model was established by autologous whole blood injection into the right basal ganglia in rats. The primary cultured microglia were treated with oxygen‐hemoglobin (OxyHb) to mimic ICH in vitro. In this experiment, specific siRNA for Tim‐3 and recombinant human TIM‐3 were exploited both in vivo and in vitro.

**Results:**

Tim‐3 was increased in the brain after ICH, which mainly distributed in microglia, but not neurons and astrocytes. However, the blockade of Tim‐3 by siRNA markedly reduced secretion of inflammatory factors, neuronal degeneration, neuronal cell death, and brain edema. Meanwhile, downregulation of Tim‐3 promoted the transformation of microglia phenotype from M1 to M2 after ICH. Furthermore, upregulation of Tim‐3 can increase the interaction between Tim‐3 and Galectin‐9 (Gal‐9) and activate Toll‐like receptor 4 (TLR‐4) pathway after ICH. Increasing the expression of Tim‐3 may be related to the activation of HIF‐1α.

**Conclusion:**

Tim‐3 may be an important link between neuroinflammation and microglia polarization through Tim‐3/Gal‐9 and TLR‐4 signaling pathways which induced SBI after ICH.

## 1 BACKGROUND

Intracerebral hemorrhage (ICH) is an acute central nervous system (CNS) disease with high mortality and disability, accounting for ~15% of all patients with stroke.^1^ Although a large number of researches have been performed to investigate the mechanisms of brain injury after ICH, there is still no effective clinical treatment which can significantly improve the prognosis of patients with drug.^2^ ICH triggers a series of complex physiological and pathological events that ultimately lead to brain damage, especially in the tissues around the hematoma.^3^, ^4^ These events include the hematoma mass effect and the potential hematoma expansion, oxidative stress, inflammatory cell infiltration, cell necrosis and apoptosis.^5^, ^6^ Previous studies reported that inflammation is closely related to brain injury after ICH and suggested that inflammation may be an effective indicator of the outcome and prognosis of ICH.^3^, ^4^ Recent studies are focusing on the effects of inflammation in brain injury after ICH. However, the mechanism is still not fully illustrated.

T‐cell immunoglobulin‐ and mucin‐domain‐containing molecule family (Tims family) is initially found in the clonal mouse asthma model.^8^ The Tims family includes eight members, and Tim‐1, Tim‐3, and Tim‐4 are predominantly expressed in the human body. Tim‐3 is an important member of the Tims family which is expressed explicitly in CD4^+^ Th1 cells and other related immune cells except Th2 cells.^9^ In addition, Tim‐3 is expressed in tissues and organs which are involved in innate immune, and specific immune response and is closely related to the progression and outcome of various diseases.^9^, ^10^ Tim‐3 is mainly expressed in microglia in the brain and involved in the inflammatory response in CNS diseases, including ischemic stroke, multiple sclerosis, and cerebral parasitic disease.^10^, ^11^

Recent studies showed that microglia/macrophages are the primary immune defender of the CNS.^4^, ^14^, ^15^ After ICH, a large number of endogenous activated microglia and exogenous macrophages rapidly gathered in brain tissues surrounding the hematoma and released inflammatory factors.^16^, ^17^ Previous studies suggested that microglia participated in brain injury through transforming into different phenotypes and releasing a large number of cytokines in the local microenvironment after ICH.^15^, ^17^ Microglia/macrophages are divided into two phenotypes, pro‐inflammatory phenotype (M1) and antiinflammatory phenotype (M2) after ICH. M1 phenotype microglia promote inflammation, while M2 phenotype microglia inhibit inflammatory response and participate in tissue reparations after ICH.^17^ The activation and polarization of microglia in the brain after ICH have been reported,^18^, ^19^ but whether Tim‐3 is involved in this remains unclear.

Galectin‐9 (Gal‐9) is one of the ligands of Tim‐3, which regulates inflammatory response in diverse diseases.^10^, ^20^, ^21^ Interaction of Tim‐3 with Gal‐9 is essential in the induction of autoimmune diseases by regulating secretion of inflammatory factors.^9^ Toll‐like receptors (TLRs) are the classical family of molecules which involved in innate immunity.^22^ TLR‐4 is an essential member of the TLRs family, and it is closely related to the inflammatory response of CNS diseases.^23^ Meanwhile, the interaction of TLR‐4 and Tim‐3 plays an essential role in the regulation of inflammation. These studies suggested that Gal‐9/Tim‐3 and TLR‐4 signaling pathways may be the potential mechanisms of neuroinflammation. However, the mechanism of Tim‐3 in inflammation of SBI after ICH it is still unclear.

In this study, we explored the relationship between Tim‐3 and ICH, particularly the function of Tim‐3 in the activation or polarization of microglia and the effect of Tim‐3 in brain injury after ICH. Thus, we investigated the expression level and potential effects of Tim‐3 in inflammation of SBI after ICH.

## 2 METHODS

### 2.1 Ethics and experimental animals

Sprague‐Dawley (SD) rats weigh 250‐300 g and about 8 weeks old, which were provided by the Shanghai Experimental Animal Center of the Chinese Academy of Sciences. All animals were fed ad libitum and housed in a quiet environment (indoor temperature about 18‐22°C). Additionally, we strived as much as possible to minimize animals’ number and their suffering.

### 2.2 Establishment of ICH model in vivo and in vitro

**Figure 1:**
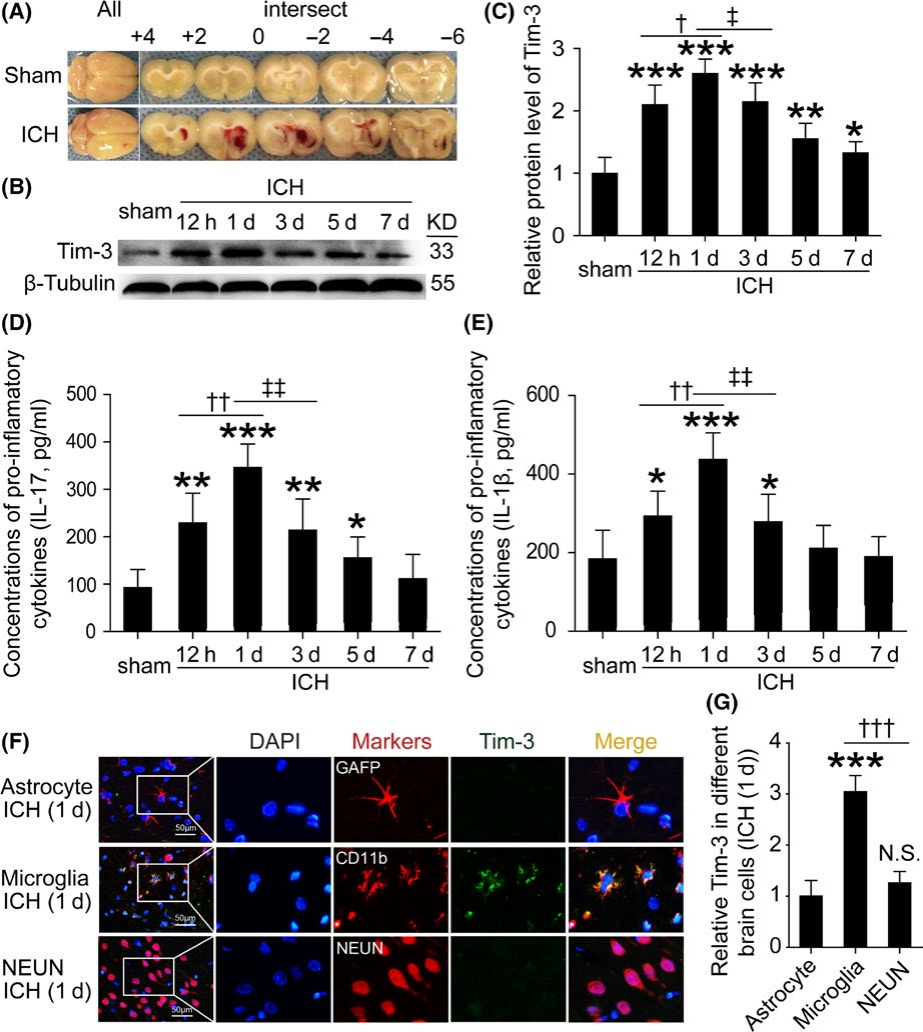
Intracerebral hemorrhage (ICH) model and levels of Tim‐3 in brain tissues which were mainly located in microglia and the levels of inflammatory factors after ICH. A, Whole brain and the largest coronal section of hematoma. B, C, Western blot analysis showed the protein levels of Tim‐3 at various time points in brain tissues after ICH. D, E, ELISA assay was used to detect the brain tissues of IL‐1β and IL‐17. F, Double immunofluorescence analysis was performed with Tim‐3 (green) and astrocytic marker GFAP, microglia marker CD11b, or neurotic maker NeuN (red) in brain sections. Nuclei were fluorescently labeled with DAPI (blue). Scale bar=50 μm. G, The relative Tim‐3 fluorescent intensity in different brain cells was shown. Data are mean ± SD. Except for (G), the rest of the graph, *P < 0.05 vs sham group; **P < 0.01 vs sham group; ***P < 0.001 vs sham group, n = 6; ^†^ *P* < 0.05 vs 12 h ICH group; ^††^ *P* < 0.01 vs 12 h ICH group, n = 6; ^‡^ *P* < 0.05 vs ICH (1 d) group; ^‡‡^ *P* < 0.01 vs ICH (1 d) group, n = 6; (G), ***P < 0.001 vs astrocyte group, NS, no significant differences, ^†††^ *P* < 0.001 vs microglia group, n = 6

In vivo ICH model was established in SD rats as described in a previous study.^24^ A schematic illustration of the coronal section of the brain is shown in Figure 1A. In vitro, primary microglia‐enriched cultures were prepared from the brain tissues of 1‐day‐old pups (from pregnant SD rats) according to our previous study.^25^ A detailed description of this method is provided in the Data Supplement.

### 2.3 Experimental grouping

The experiments were divided into three parts (experiment I‐III), for details, please see the online‐only Data Supplement.

### 2.4 Cell nuclear protein and cytoplasm protein extraction

Cell nuclear protein and cytoplasm protein extraction of brain tissues were performed by using a Cell nuclear protein and cytoplasm protein extraction kit (BeyoTime Institute of Biotechnology, Nantong, China). All the reagent preparation and experimental operation were performed according to the manufacturer’s instructions. Briefly, the following some steps were performed: the entire experimental procedure was carried out on ice or at 4°C. (a) Shred the organization as much as possible. The tissue homogenate buffer was formulated by adding appropriate amounts of cytoplasmic protein extraction buffer A and B and PMSF. Mix tissue and tissue homogenate buffer; (b) After homogenization, the mixed slurry was transferred to a plastic centrifuge tube and placed on ice for 15 minutes; (c) Centrifuge at 1500 *g* for 5 minutes at 4°C. The supernatant was transferred to a precooled plastic tube to extract a portion of the cytoplasmic protein; (d) Add 50 μL of nuclear protein extraction reagent to precipitation and shake it for 15‐30 seconds, shaking once every 2 minutes for 30 minutes. (e) Centrifuge at 12 000‐16 000 *g* for 10 minutes at 4°C, and the supernatant was the nuclear protein.

### 2.5 Western blot analysis

Western blot analysis was performed as described previously.^26^ For details, please see the online‐only Data Supplement.

### 2.6 ELISA

The concentration of brain tissue of IL‐1β and IL‐17 were determined by ELISA using the rat IL‐1β and IL‐17 kits (Cloud Clone Corp; SEA563Ra, SEA063Ra, Wuhan, China). This assay was performed according to the manufacturer’s instructions, and the data were expressed relative to a standard curve prepared for IL‐1β and IL‐17.

### 2.7 Short‐term and long‐term neurological functions

In experiment II, we tested neuro‐functional impairment of rats with a previously published scoring system which monitors their activity, appetite, and neurological deficits at 72 hours after ICH.^27^ Assessments of sensorimotor deficits will be performed before and after ICH at day 1, 3, 5, 7, 14, 21, and 28 with the adhesive removal and foot‐fault test follow the standard method.^28^, ^29^

### 2.8 Immunofluorescence analysis

Immunofluorescence analysis was performed as described previously.^30^ For details, please see the online‐only Data Supplement.

An observer who was blind to the experimental group performed the quantitative analysis. Staining images were auto‐thresholded using Image J program (NIH, Bethesda, MD, USA) to subtract background staining. Relative Tim‐3 fluorescence intensity in different brain cells at 1 day after ICH was measured by the ratio of the fluorescence intensity of each group to the fluorescence intensity of the astrocyte. The fluorescence intensity in each cell area was calculated. ROI were selected within the ipsilateral around the hematoma.

The number of M1 microglia/macrophages (CD16/32+/CD11b+ cells) or M2 microglia/macrophages (CD206+/CD11b+ cells) at 1 day after ICH was counted from one microscopic field randomly selected around the hematoma area (within 300 μm to the hematoma).

### 2.9 Transfection of rhTIM‐3 and siRNA in vivo and in vitro

The drilling site of the intracerebroventricular region for rats was determined as described in previous studies.^31^, ^32^ The relevant dosage of recombinant human TIM‐3 (rhTIM‐3) and siRNA for intracerebroventricular injection was in accordance with manufacturer instructions.

To knockdown Tim‐3, specific siRNAs against Tim‐3 were obtained from Guangzhou Ribo Biotechnology Co., Ltd. (Guangzhou, China). To ensure the knockdown efficacy, we mixed the three target sequences for the following studies. The three target sequences for siRNA design are shown below.

1. GCAGGATTTGAGTCTTATT
2. GGTGGCATATGCTTAACAT
3. GCATGAGGAACCTGAGTTT

Tim‐3 siRNA sequences were dissolved in RNase‐free water to the concentration of 500 pmol/10 µL and then diluted by the same volume of transfection reagent. Four microgram of nucleic acid was injected intracerebroventricularly per rat.

### 2.10 Fluoro‐Jade B staining and TUNEL staining

Fluoro‐Jade B (FJB) and TUNEL staining was performed as described previously.^7^ For details, please see the Supplementary Material.

### 2.11 Brain water content measurement

Brain tissue water content (%) is calculated as [(WW − DW)/WW] × 100. For details, please see the online‐only Data Supplement.

### 2.12 Immunoprecipitation analysis

Immunoprecipitation tests were performed as reported previously.^7^ For details, please see the online‐only Data Supplement.

### 2.13 Statistical analysis

All data were presented as mean ± SD. Graph pad prism 7.0 was used for all statistical analysis. Data groups (two groups) with normal distribution were compared using the two‐sided unpaired Student test. Differences in means among multiple groups were analyzed using one‐way ANOVA or two‐way ANOVA followed by the Bonferroni/Dunn post hoc test. Behavioral tests use the two‐way repeated ANOVA *P* < 0.05 was considered statistically significant.

## 3 RESULTS

### 3.1 General observation

The body temperature, mean arterial pressure, and body weight of rats in each experimental ICH group did not change significantly (data not shown). The mortality rate of rats in the normal and sham group was 0% (0/24 rats), and it was 5.9% (6/102 rats) in all experimental ICH groups.

### 3.2 Tim‐3 was increased and mainly in microglia after ICH

To detect the expression level of Tim‐3 in brain tissues after ICH, we tested the protein samples from brain tissues around the hematoma by Western blot analysis. Comparing with the sham group, the level of Tim‐3 was increased significantly from 12 hours after ICH, reached the peak point at 24 hours, and then reduced gradually after that (Figure 1B,C). Also, ELISA results showed that the levels of IL‐1β and IL‐17 in brain tissues were significantly increased at 24 hours after ICH and then decreased gradually to the level of the sham group at 7 days (Figure 1D,E). To further clarify the cell type which expressed Tim‐3, we detected the expression levels of Tim‐3 in astrocytes, microglia, and neurons, respectively. Double immunofluorescence staining was performed on brain sections which incubated by Tim‐3 and GFAP (astrocytic marker), CD11b (microglia marker), or NeuN (neuronal marker), respectively. We observed a marked increment of Tim‐3 in microglia, but not in neurons and astrocytes at 1 day after ICH (Figure 1F,E; Figure S4A,B).

### 3.3 The secretion of IL‐1β and IL‐17 was blocked by inhibiting Tim‐3 in the brain after ICH

**Figure 2:**
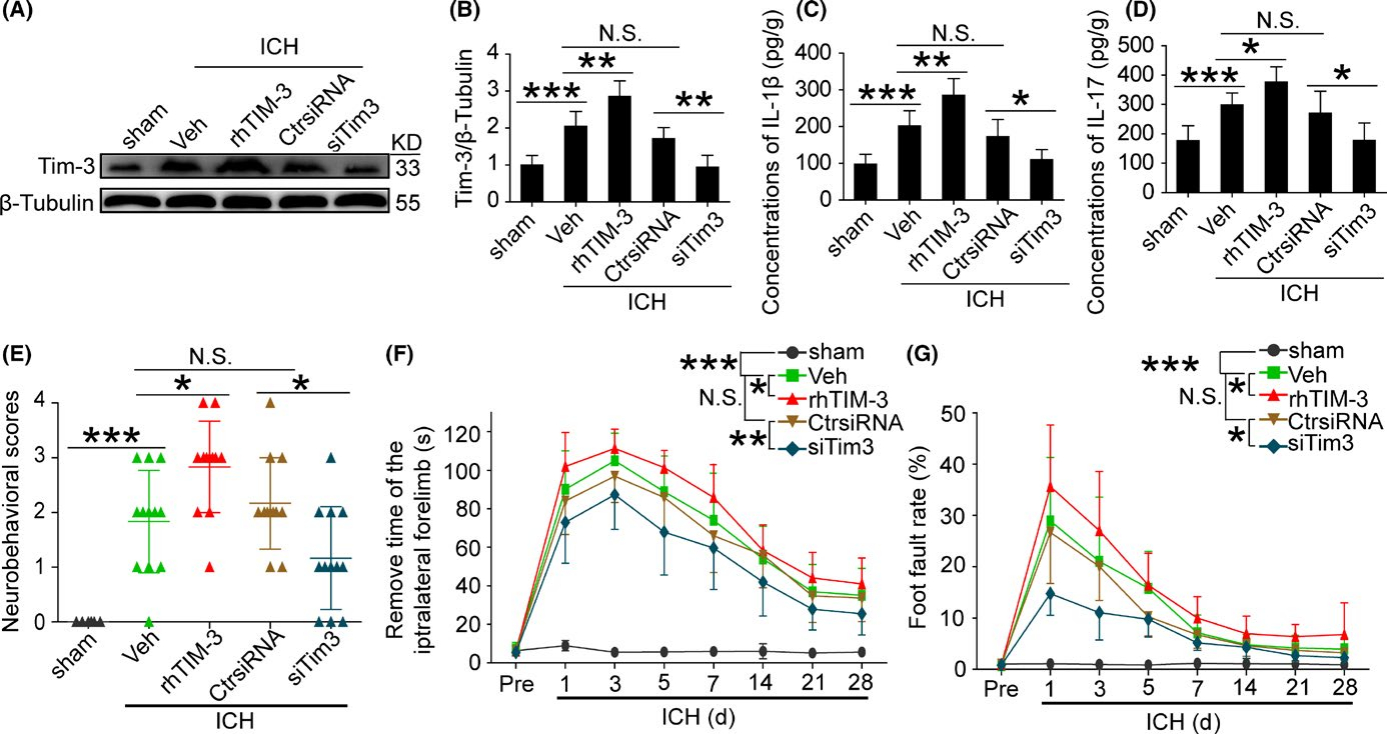
Effects of rhTIM‐3 and Tim‐3 siRNA treatments on the protein level of Tim‐3 in the brain tissues of IL‐1β and IL‐17 under ICH conditions. A and B, Western blot analysis showed the protein level of Tim‐3 in brain tissues in various groups. C and D, ELISA assay was used to detect the brain tissues content of IL‐1β and IL‐17 at 1 d after ICH. Data are mean ± SD. NS: no significant differences; *P < 0.05; **P < 0.01; ***P < 0.001, n = 6. E, Neurological behavior scores. F, Adhesive removal test. G, Foot‐fault test. Data are mean ± SD. NS: no significant differences; *P < 0.05; **P < 0.01; ***P < 0.001, n = 6. rhTIM‐3, recombinant human TIM‐3

To further define the role of Tim‐3 in the secretion of IL‐1β and IL‐17 in brain tissues after ICH, the rhTIM‐3 and Tim‐3 siRNA were applied to regulate the levels of Tim‐3 in this study. Consistent with the results above, Western blot analysis showed Tim‐3 expression level was higher at 1 day after ICH than that in the sham group, while it was significantly increased by rhTIM‐3 treatment and decreased by Tim‐3 siRNA treatment (Figure 2A,B). In ELISA analysis, we accidentally discovered that the level of IL‐1β and IL‐17 in ICH rat brain tissue was promoted at 1 day after ICH, which was significantly decreased by Tim‐3 siRNA treatment and increased by rhTIM‐3 treatment (Figure 2C,D).

### 3.4 Inhibition of Tim‐3 improved short‐term and long‐term neurological functions after ICH

To identify the impact of Tim‐3 on neurological behavior at day 3 after ICH, behavioral activity was performed. Neurological behavior was severely impaired in rhTIM‐3 group compared with the ICH group, which was dramatically attenuated in Tim‐3 siRNA group (Figure 2E). To investigate the effect of Tim‐3 in long‐term neurological outcomes, two independent behavior tests: adhesive test and foot‐fault test were performed until day 28 to measure neurological function. After ICH, the vehicle‐treated group showed spontaneous recovery, rhTIM‐3‐treated rats had a dramatically worse performance in adhesive tests than the vehicle group, and siTim‐3 rats showed better performance in adhesive test compared with Ctr‐siRNA group (Figure 2F). In addition, siTim‐3 rats performed significantly better throughout day 28 compared with Ctr‐siRNA group as shown in the foot‐fault test (Figure 2G).

### 3.5 Tim‐3 knockdown by siRNA mitigated brain injury after ICH

**Figure 3:**
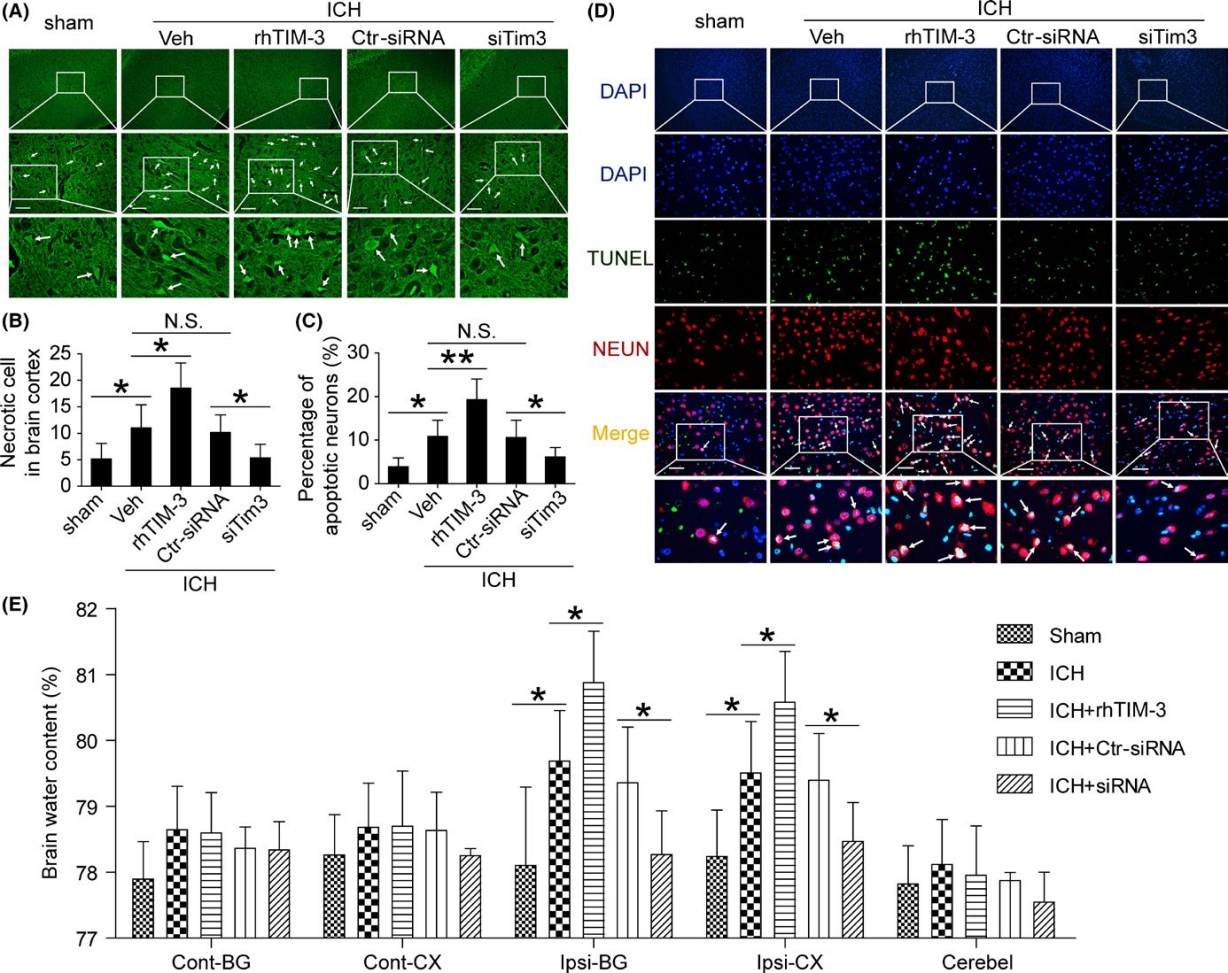
Effects of Tim‐3 siRNA on neuronal degeneration and cell death, and brain water content under ICH conditions. A, Fluoro‐Jade B (FJB) staining (green) shows neuronal degeneration in the cerebral cortex. Scale bar = 100 μm. B, Degeneration of neuronal cells in brain tissues is shown. C, Percentage of apoptotic neurons is shown. D, Double immunofluorescence for NeuN (red) and TUNEL (green) counterstained with DAPI (blue) was performed. Arrows point to NeuN/TUNEL‐positive cells. Scale bar = 100 μm. E, Bar graphs showing the effects of rhTIM‐3 and Tim‐3 siRNA on brain water content. Cont: contralateral; Ipsi: ipsilateral; CX: cortex; BG: basal ganglia; Cerebel; cerebellum. Data are mean ± SD. NS: no significant differences; *P < 0.05; **P < 0.01, n = 6. rhTIM‐3, recombinant human TIM‐3

To detect the effects of Tim‐3 in ICH‐induced SBI, the FJB and TUNEL staining were performed to test neuronal degeneration and death in the brain in all groups. Comparing with the sham group, the number of FJB‐positive cells was increased in brain tissues in the ICH group, which was significantly aggravated by rhTIM‐3 treatment and attenuated by Tim‐3 siRNA treatment (Figure 3A,B). TUNEL‐positive cells exhibited similar results as FJB‐positive cells (Figure 3C,D). In addition, brain water content was higher in the ICH group than that in the sham group. The brain water content was higher in ICH rats with rhTIM‐3 treatment than that in the ICH control group, while the brain water content was lower in ICH rats with Tim‐3 siRNA treatment than that in the ICH group (Figure 3E). These data indicated that the inhibiting Tim‐3 with siRNA could reduce the ICH‐induced SBI.

### 3.6 Tim‐3 induced polarization of microglia in the brain after ICH

**Figure 4:**
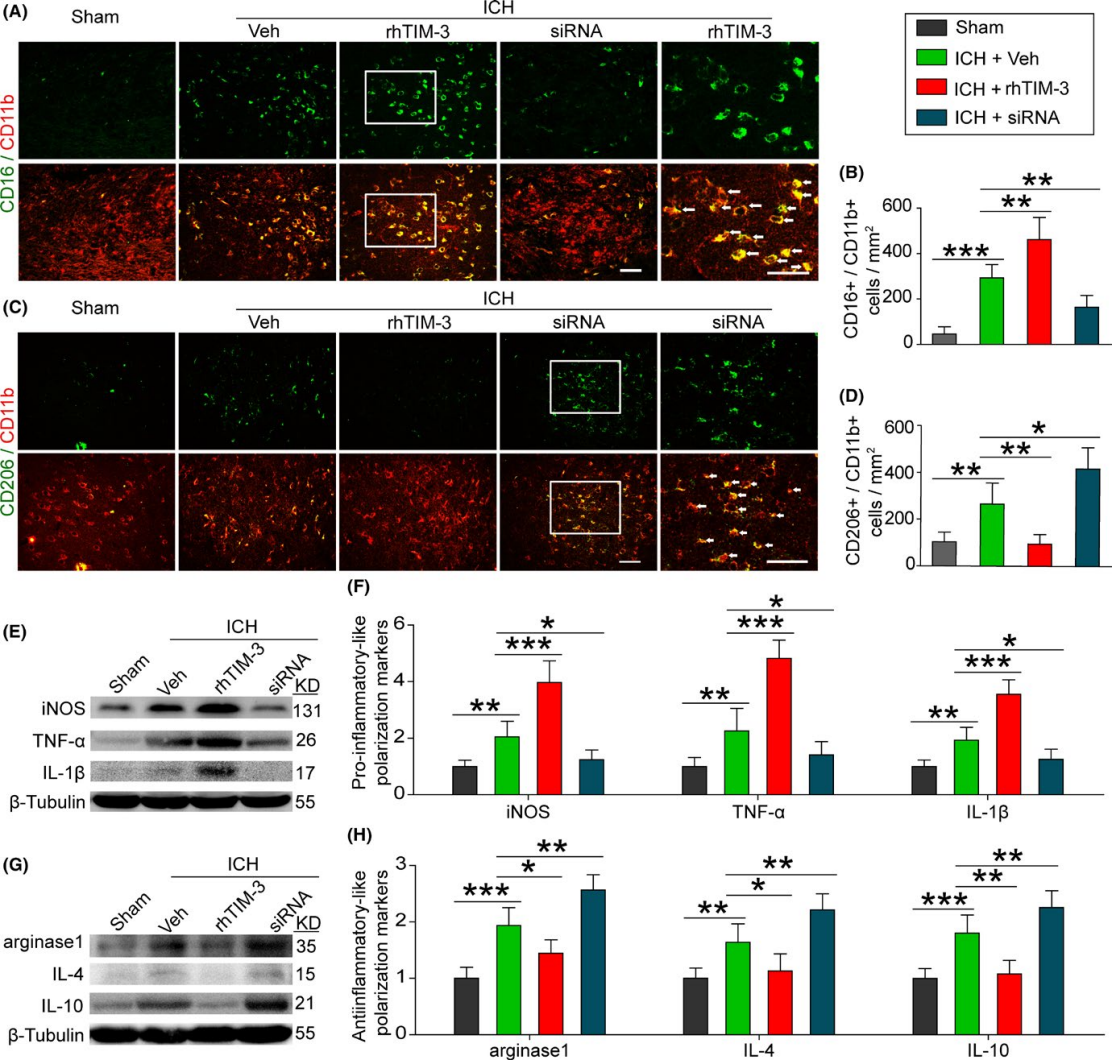
Effects of Tim‐3 on ICH‐induced microglia polarization and changes of phenotype. Sections were stained for CD16/CD11b (pro‐inflammatory microglia marker) or CD206/CD11b (antiinflammatory microglia marker). Representative images were shown in (A) and (C) and percentage of CD16‐positive cells or CD206‐positive cells was shown in (B) and (D). Scale bar = 50 μm. Data are mean ± SD. NS: no significant differences; *P < 0.05; **P < 0.01; ***P < 0.001, n = 6. E and G, The immunoblots showed TNF‐α, IL‐1β, iNOS, arginase1, IL‐4, and IL‐10 produced by microglia under indicated treatment. F and H, The quantitative analyses of TNF‐α, IL‐1β, iNOS, arginase1, IL‐4, and IL‐10 in the immunoblots. Data are mean ± SD. NS: no significant differences; *P < 0.05; **P < 0.01; ***P < 0.001, n = 6

Next, we observed the effects of Tim‐3 on the microglia polarization in brain tissue after ICH by using rhTIM‐3 and Tim‐3 siRNA (Figure 4A‐D). The results of double immunofluorescence staining showed that ICH‐induced microglia polarization to a pro‐inflammatory phenotype, as defined by CD16/CD11b‐positive, and also to an antiinflammatory phenotype, as defined by CD206/CD11b‐positive (Figure 4A‐D). Compared with the sham group, CD16/CD11b‐positive cells were increased in the ICH group, while it was significantly aggravated by rhTIM‐3 and attenuated by Tim‐3 siRNA treatment (Figure 4A,B). Compared with the sham group, CD206/CD11b‐positive cells were decreased in the ICH group, while it was significantly attenuated by rhTIM‐3 and aggravated by Tim‐3 siRNA treatment (Figure 4C,D).

In addition, as previously reported,^33^ the M1 and M2 macrophages are defined by single phenotypic markers (CD206 and CD16) is not sufficient to identify microglia polarization. Inflammation‐associated molecules, including pro‐inflammatory factors TNF‐α, IL‐1β, and iNOS and antiinflammatory factors arginase1, IL‐4, and IL‐10 were also tested to provide more information on the biological state of microglia after ICH (Figure 4E‐H). The results showed that the protein levels of all inflammation‐associated molecules in brain tissues were significantly increased by ICH treatment. However, the results showed that the protein levels of pro‐inflammation molecules were significantly aggravated by rhTIM‐3 and attenuated by Tim‐3 siRNA treatment, whereas the protein levels of antiinflammation molecules were significantly attenuated by rhTIM‐3 and aggravated by Tim‐3 siRNA treatment.

### 3.7 Tim‐3 knockdown inhibited ICH induced the interaction between Tim‐3 and Gal‐9 and promoted the interaction between TLR‐4 and Gal‐9

**Figure 5:**
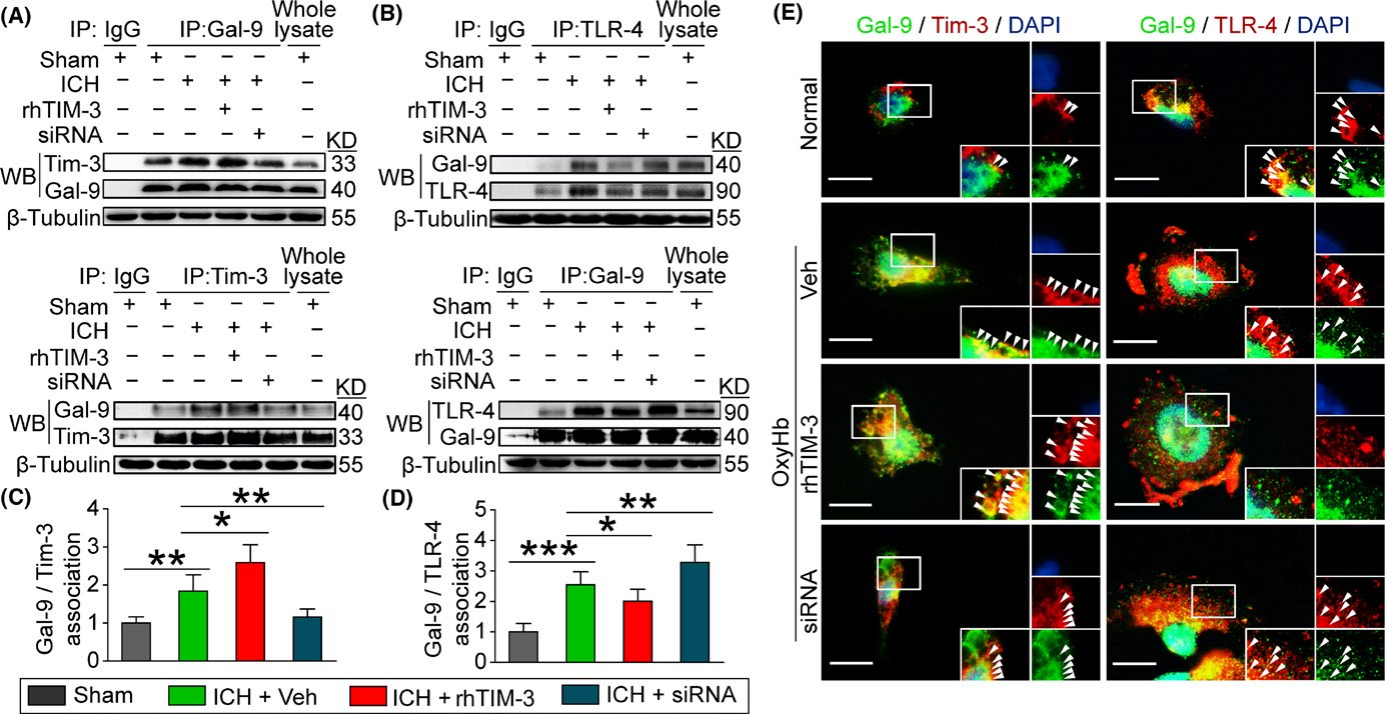
Effects of Tim‐3 on Gal‐9/Tim‐3 and Gal‐9/TLR‐4 interactions after ICH. A and B, Gal‐9/Tim‐3 interaction and Gal‐9/TLR‐4 interactions in brain tissues were determined using immunoprecipitation (IP). C and D, Quantitative analysis was performed. Data are mean ± SD. *P < 0.05; **P < 0.01; ***P < 0.001, n = 6. E, Double immunofluorescence for Tim‐3 or TLR‐4 (red) and Gal‐9 (green) counterstained with DAPI (blue) was performed. Arrows indicated the overlap of Gal‐9 and Tim‐3 or TLR‐4 around the cell edge. Scale bar = 10 μm

It has been reported that Tim‐3 interacts with Gal‐9 on the cell membrane and promotes inflammation in the CNS diseases.^21^, ^34^ However, it has not been reported that TLR‐4 interacts with Gal‐9 on the cell membrane after ICH. To further investigate the mechanism whether Tim‐3 siRNA could rescue the SBI in ICH rats, we analyzed the interaction between Tim‐3/Gal‐9 and TLR‐4/Gal‐9 by immunoprecipitation in brain tissues following ICH. The results of immunoprecipitation showed that, compared with the sham group, Tim‐3 and Gal‐9 interaction was increased in the ICH group, and it was significantly aggravated by rhTIM‐3 and attenuated by Tim‐3 siRNA treatment (Figure 5A,B). The results also showed that compared with the sham group, TLR‐4 and Gal‐9 interaction increased in the ICH group, while it was significantly attenuated by rhTIM‐3 and aggravated by Tim‐3 siRNA treatment (Figure 5C,D).

In addition, compared with the normal group, the yellow areas by arrows indicated the overlap of Tim‐3 and Gal‐9 was significantly increased in the OxyHb group, especially the distribution of Tim‐3 alongside the cell edge, which was significantly aggravated by rhTIM‐3 and attenuated by Tim‐3 siRNA treatment (Figure 5E). Compared with the normal group, the yellow areas by arrows indicated the overlap of TLR‐4 and Gal‐9 was not significantly increased in the OxyHb group, but it was significantly attenuated by rhTIM‐3 and aggravated by Tim‐3 siRNA treatment (Figure 5E).

### 3.8 The expression level of HIF‐1α protein in cytoplasm and nucleus is closely related to the regulation of Tim‐3

**Figure 6:**
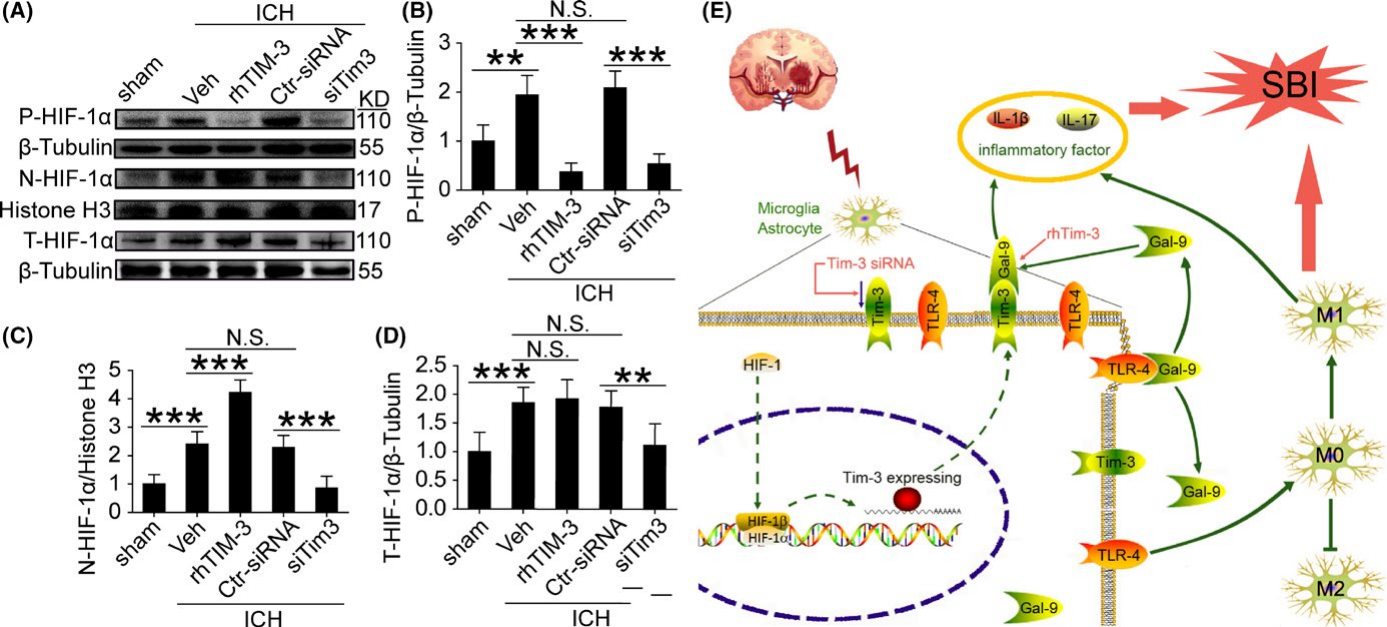
The expression level of HIF‐1α protein in cytoplasm and nucleus is closely related to the regulation of Tim‐3 after ICH. A‐D, Western blot analysis and quantification of the protein level of HIF‐1α in the cytoplasm and the nucleus. Data are mean ± SD. NS: no significant differences. Data are mean ± SD. *P < 0.05; **P < 0.01; ***P < 0.001, n = 6. Hypothesis of potential mechanisms of Tim‐3 in inflammatory signaling pathway under ICH condition. Green colored arrows indicate the ICH‐induced Tim‐3 actions, and red colored arrows indicate the effects of rhTIM‐3 and Tim‐3 siRNA. HIF‐1α transports from the cytoplasm to the nucleus, Tim‐3 expression increased after ICH. A large number of Tim‐3 interact with Gal‐9, and the results are activated Tim‐3/Gal‐9 signaling pathway which promotes the production of inflammatory factors. In addition, a large number of TLR‐4 exposure, activation of TLR‐4 signaling pathway which promotes microglia to the pro‐inflammatory state. Two inflammatory pathways are activated, leading to SBI after ICH. rhTIM‐3, recombinant human TIM‐3

To detect the expression level of HIF‐1α protein in cytoplasm and nucleus, Western blot analysis was first performed to test HIF‐1α in the subcellular space. Compared with the sham group, plasm‐protein HIF‐1α was increased in the ICH group, which was significantly attenuated by rhTIM‐3 and aggravated by Tim‐3 siRNA treatment (Figure 6A,B). Compared with the sham group, nucleoprotein HIF‐1α was increased in the ICH group, which was significantly aggravated by rhTIM‐3 and attenuated by Tim‐3 siRNA treatment (Figure 6A,C). Compared with the sham group, total protein HIF‐1α was increased in the ICH group, which was not significantly aggravated by rhTIM‐3, but it was significantly attenuated by Tim‐3 siRNA treatment (Figure 6A,D).

## 4 DISCUSSION

As previously reported, experimental and clinical results indicated that inflammation was critically involved in the pathogenesis of SBI after ICH.^3^, ^4^ The level of expression of Tim‐3 is elevated in CNS diseases, and it activates microglia, where it plays a vital role in the inflammatory processes associated with neutrophil infiltration.^14^, ^15^, ^35^ Gal‐9 as a ligand of Tim‐3 can be upregulated by the Tim‐3 and is involved in CNS diseases associated with inflammation.^10^ Recent studies suggested that Tim‐3 was constitutively expressed on cells of the innate immune system in both humans and mice, and it can synergize with TLRs.^10^ Therefore, a complete understanding of the relationship between Tim‐3, Gal‐9, and TLRs may better explain the pathophysiological mechanisms of SBI after ICH.

Our study showed the effects of Tim‐3 on the pathogenesis of SBI in a rat ICH model. In experiment I, the results showed that the higher level of Tim‐3 expression in microglia in brain tissues in SBI after ICH. It was shown that the most significant time point was 1 day after ICH. In experiment II, firstly, we found that the usage of Tim‐3 siRNA eliminated ICH‐induced Tim‐3 upregulation, while rhTIM‐3 showed the opposite effect. Secondly, Tim‐3 siRNA treatment decreased the secretion of inflammatory factors in the brain after ICH, improved long‐term neurological outcomes as well as the neuronal degeneration and death, and ameliorated brain edema after ICH. Finally, Tim‐3 siRNA treatment induced microglia polarization to an antiinflammatory phenotype (M2) and the levels of antiinflammation factors were increased, while rhTIM‐3 showed the opposite effect. In experiment III, the results were shown as follows: Tim‐3 siRNA treatment rescued ICH‐induced disruption in the interaction between Tim‐3 and Gal‐9 and promoted the interaction between TLR‐4 and Gal‐9. Besides, increased protein levels of Tim‐3 induced by ICH may be closely related to HIF‐1α from the cytoplasm to the nucleus (Figure 6). Based on these results, we hypothesized here that high‐level expression of Tim‐3 binds closely to Gal‐9, the activation of the Tim‐3/Gal‐9 signaling pathway promoted the production of pro‐inflammatory factors. At the same time, TLR‐4 lost its association with Gal‐9, thus causing more TLR‐4 exposure and the activation of TLR‐4 signaling pathway which promoted microglia transform to pro‐inflammatory state (M1 phenotype). Therefore, we can conclude that there are two pathways promoting inflammation involved in SBI after ICH. The mechanisms of this study were shown in Figure 6E.

Gal‐9 was thought to be essential for regulating cell homeostasis and inflammation. Recent studies demonstrated that Gal‐9 induced various biological reactions, such as cell aggregation, adhesion, activation, and apoptosis.^36^, ^37^ Gal‐9 showed immunomodulatory properties by inducing Th1 cell (not Th2 cell) death through interaction with Tim‐3.^38^ In addition, Tim‐3 and Gal‐9 were also expressed in brain tissues and involved in inflammation in the rat stroke model.^21^ High‐level expression of Tim‐3 in glial cells triggers and activates the Tim‐3/Gal‐9 signaling pathway and promotes the release of pro‐inflammatory factors, but its role in ICH is unknown. Our study demonstrated that the expression level of Gal‐9 was upregulated together with the increasing of Tim‐3 on microglia after ICH. Additionally, we used rhTIM‐3 to validate the effect of Tim‐3/Gal‐9 signaling pathway and Tim‐3 siRNA to disrupt the effect of Tim‐3/Gal‐9 signaling pathway on SBI after ICH. These results suggested that blocking Tim‐3/Gal‐9 signaling pathways may rescue SBI and reduce the release of inflammatory factors in brain tissues after ICH.

TLR‐4, a major member of the Toll‐like receptors, which involved in SBI and neurobehavioral dysfunction through TLR4‐mediated inflammatory pathway after experimental subarachnoid hemorrhage.^23^ In addition, the TLR‐4 signaling pathway has been widely studied in macrophages polarization and its activation is closely related to M1 macrophage produces.^39^, ^40^ For various neurological diseases, such as traumatic brain injury, multiple sclerosis, or ischemic stroke, M1 macrophages/microglia are generally to exacerbate neuronal necrosis and inflammation, whereas M2 macrophages/microglia inhibit inflammation and are beneficial for tissue reparations.^4^, ^41^ Our results confirmed that the high‐level expression of Tim‐3 after ICH caused TLR‐4 separate from Gal‐9; thus, TLR‐4 was exposed. The exposure of TLR‐4 may promote microglia transfer to the M1 phenotype and release more pro‐inflammatory factors, while TLR‐4 binds closely to Gal‐9 which may promote microglia to the M2 phenotype and release more antiinflammatory factors. These results suggested that blocking the TLR‐4 signaling pathway may reduce SBI and induce microglia transfer to antiinflammatory phenotype in the brain after ICH.

In this study, we investigated the mechanism of SBI after ICH through the Tim‐3/Gal‐9 signaling pathway and TLR‐4‐mediated inflammatory responses, which were closely related to the increasing level of Tim‐3. However, the reason for the high‐level expression of Tim‐3 after ICH is unclear. Previous studies have shown that HIF‐1α may induce the inflammatory cells into the hypoxic brain tissues by regulating the level of glial Tim‐3 and HIF‐1α genes which are associated with metabolic responses.^35^ We detected the distribution of HIF‐1α in the cytoplasm or nucleus or the whole cell after ICH, and the results showed that the level of HIF‐1α levels increased in cytoplasm and nucleus after ICH; the level of HIF‐1α decreased in cytoplasm and the level of HIF‐1α increased in nucleus after rhTIM‐3 treatment; the level of HIF‐1α in nucleus and cytoplasm decreased after Tim‐3 siRNA treatment. Therefore, we speculate that elevated the level of Tim‐3 after ICH may be closely related to the different expression of HIF‐1α in cytoplasm and nucleus. However, the above results do not demonstrate that elevated levels of Tim‐3 in brain tissue are closely related to the transport of HIF‐1α from the cytoplasm to the nucleus. In order to confirm the relationship between Tim‐3 and HIF‐1α, further research is needed in the future.

There are several limitations to this study. In vitro experiments, we showed OxyHb might increase the expression of Tim‐3. However, there is no study of other contents of hematoma. So this study cannot explain all the mechanism in the high level of Tim‐3 expression post‐ICH. Lastly, we did not figure out it was the Tim‐3/Gal‐9 signaling pathway or the TLR‐4‐mediate inflammation pathway that played the dominant role.

## 5 CONCLUSIONS

The present study demonstrated that the level of Tim‐3 was increased after ICH and mainly distribute in microglia. The regulation of Tim‐3 expression was performed by rhTIM‐3 and Tim‐3 siRNA, which aggravated or relieved, respectively, in SBI after ICH. High‐level expression of Tim‐3 induced the activation of two inflammatory pathways that all aggravated SBI after ICH. The activation of Tim‐3/Gal‐9 signaling pathway promoted the release of inflammatory factors; on the other hand, the activation of the TLR‐4 signaling pathway is closely related to microglia transformation to M1 phenotype. Tim‐3 may be an important link between neuroinflammation and the polarization of microglia, and negative regulation of glial Tim‐3 signal may be a novel therapeutic target for ICH.

## CONFLICT OF INTEREST

The authors declare no conflict of interest.
