# Supplementary material for: Negative regulation of glial Tim‐3 inhibits the secretion of inflammatory factors and modulates microglia to antiinflammatory phenotype after experimental intracerebral hemorrhage in rats
Source: CNS Neurosci Ther. 2019 Jan 24;25(6):674–84. doi: 10.1111/cns.13100 (PMC6515709; doi:10.1111/cns.13100)
Supplement: Supplementary file 1 [file CNS-25-674-s001.docx]

**Title:** **Negative regulation of glial Tim-3 inhibits secretion of inflammatory factors and modulates microglia to anti-inflammatory phenotype after experimental** **intracerebral hemorrhage in rats.**

Zhou-Qing Chen^1^, Hao Yu^2^, Hai-Ying Li^1^, Hai-Tao Shen^1^, Xiang Li^1^, Ju-Yi Zhang^1^, Zhu-Wei Zhang^1^, Zhong Wang^1^, Gang Chen^1^

1. **Establishment of ICH model *in* *vivo* and** ***in vitro***
   1. ***In* *vivo***

Briefly, rats were anesthetized with 10% chloral hydrate (4 ml/kg, i.p.). After successful anesthesia, rats were fixed in brain stereotactic instrument (Anhui Zhenghua Biological Equipment Co. Ltd., China). According to the rat head stereotactic anatomical map [^1^](#_ENREF_1), we determined the drilling position (coronal and sagittal midline at the intersection of 0.2 mm, sagittal right 3.5 mm), for the right basal ganglia opened window diameter of about 2 mm and 100µl of autologous arterial blood was injected into the basal ganglia at a rate of 20 µl/min. The micro-syringe needle stagnated for 10 min and then slowly withdrawn after injection and sterile bone wax closed the hole in the skull. The rats in the sham group received basal ganglia puncture but did not inject autologous arterial blood. During the establishment of the model, the heart rate, blood pressure, and body temperature were monitored in real time, and the rectal temperature was maintained at 37±0.5℃.

***1.2 In vitro***

Primary microglia-enriched cultures were prepared from the brain tissues of 1-day-old pups (from pregnant SD rats) according to our previous study [^2^](#_ENREF_2). Firstly, the 1-day-old pups were cut off from the head, the scalp and skull are opened, and the brain tissues were collected and removed brain stem and cerebellum. After removing the arachnoid tissue and blood vessels of the brain surface, brain tissues were placed in serum-free DMEM/F12 medium. Next, 0.125% trypsin (with EDTA) was added to the medium, and the brain tissues were digested at 37°C for 5 min. The digestion was terminated by washing tissue with PBS 3 times. Secondly the brain tissue suspension was centrifuged at 500g for 5min, and the pellet was suspended in fresh DMEM/F12 medium containing 10% fetal bovine serum, 1 mM sodium pyruvate, 2 mM L-glutamine, 100 mM nonessential amino acids, 50 U/ml penicillin, and 50mg/ml streptomycin (All from GIBCO, Carlabad, CA, USA). Finally, cells were seeded in the 150-cm^2^ culture flask with fresh medium. Afterward, the full medium was changed half every two days. Generally, rough 2 weeks after initial seeding; a confluent monolayer of glial cells was achieved. Microglia were separated from astrocytes by shaking the flask for four h at 150 rpm and collected by centrifugation and re-seeded in 12-well plates with fresh DMEM/F12 medium.

1. **Experimental grouping**

In experiment 1, 36 rats (38 rats were used and 36 rats survived after the surgery) were randomly assigned into 6 groups (n = 6 in each group): sham group, and 12 hours, 1, 3, 5 and 7 days after ICH. The rats were sacrificed at 1d after operation in the sham group and were sacrificed at respective time points (12 h, 1 d, 3 d, 5 d, and 7 d) in the ICH group (Fig. S1).

**Supplemental Figure 1**


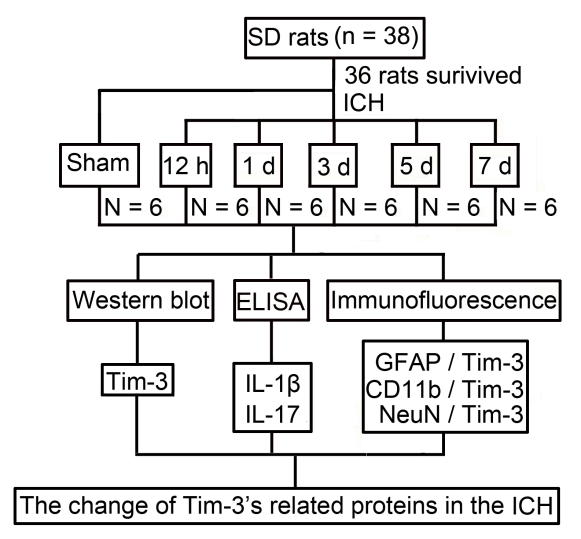


In experiment II, 144 rats (148 rats were used and 144 rats were survived after the surgery) were randomly divided into 5 groups: Sham, ICH + Veh, ICH + rhTim-3, ICH + Ctr-SiRNA, and ICH + Tim-3 SiRNA group (n=24 for sham group, n = 30 for other groups). The rats in the ICH+rhTim-3 group received immediately rhTim-3 by intracerebroventricular injection after ICH. At two days before ICH onset of successful operation, the rats in the ICH+Ctr-SiRNA group were injected with Ctr-SiRNA, and the rats in the ICH+SiRNA group were injected with Tim-3 SiRNA. At 24 hrs or 28 days after ICH, rats were sacrificed and the brain tissues were collected for various experimental analyzes (Fig. S2). In experiment II, primary cultured microglia were exposed to 20 μM OxyHb to mimic ICH *in vitro* and partitioned into 4 groups: control, OxyHb, OxyHb+Tim-3 SiRNA, and OxyHb+rhTim-3 group.

**Supplemental Figure 2**


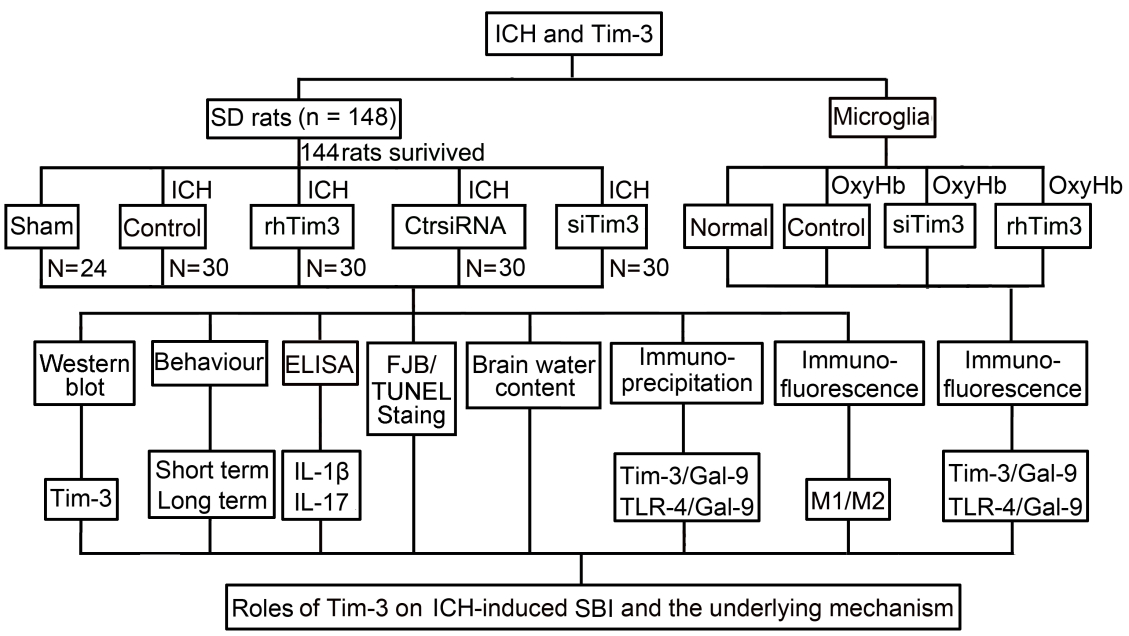


In experiment III, based on experiment II, we extracted the cytoplasm, nucleus, and total protein, and detected the expression of HIF-1α in different intervention groups by Western blot. This experiment was conducted to determine whether the levels of Tim-3 were associated with HIF-1α cell distribution after ICH (Fig. S3).

**Supplemental Figure 3**

**
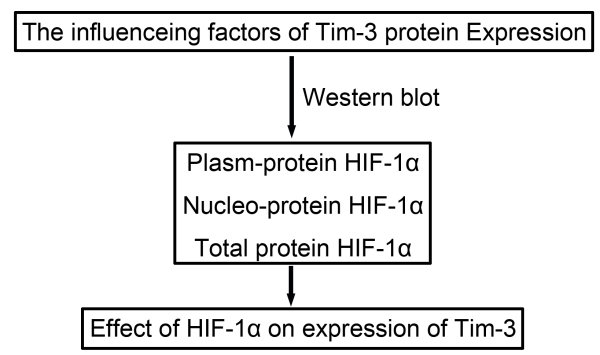
**

1. **Western blot analysis**

Western blot analysis was performed as described previously.[^3^](#_ENREF_3) Briefly, the brain samples were cut and dissected using a brain chisel and mechanically lysed in the mixture lysis buffer with phenylmethylsulfonyl fluoride (PMSF). The protein concentrations were measured by the bicinchoninic acid (BCA) method using enhanced BCA protein assay kit (all from BeyoTime Institute of Biotechnology, China). Protein samples (20 μg/lane) were loaded on a 12% SDS-polyacrylamide-gel, separated, and electrophoretically transferred to a polyvinylidene difluoride membrane (PVDF, Millipore, USA), which was blocked with 5% bovine serum albumin (BSA, Biosharp, China) for 1h at room temperature. The membrane was then incubated overnight at 4°C with primary antibodies. The primary antibodies against Tim-3, HIF-1α, TLR-4, Gal-9, Histone H3, Arginase-1, iNOS, IL-10, and IL-4 were used in this experiment and all purchased from Abcam (USA). Besides, the β-Tubulin was also detected and served as loading control. PVDF membrane was incubated with the corresponding HRP-conjugated secondary antibodies and the bands signal were detected by using enhanced chemiluminescence (ECL) kit. Image J software (NIH, USA) was used to analyze the optical density of bands.

1. **Immunofluorescence analysis**

For *in vivo* experiments, the brain samples were fixed in 4% paraformaldehyde, embedded in paraffin, cut into 4μm sections, which was dewaxed immediately before immunofluorescence staining. For *in vitro* experiments, microglia were fixed in 4% paraformaldehyde after various treatments. Then, sections and microglia were stained with primary antibodies (Tim-3, GFAP, NeuN, CD11b, CD16, CD206, TLR-4, and Gal-9 were used in this experiment and all purchased from Abcam, USA) and appropriate secondary antibodies. Normal rabbit IgG was used as a negative control (data not shown). Finally, sections and microglia were observed by a fluorescence microscope (OLYMPUS BX50/BX-FLA/DP70, Japan) or a laser scanning confocal microscope (ZEISS LSM 880, Germany). At least six random sections from each sample were examined, and representative results were shown. The relative fluorescence intensity was analyzed with the Image J program (NIH, USA). The quantitative analysis was performed by an observer who was blind to the experimental group.

An observer who was blind to the experimental group performed the quantitative analysis. Staining images were auto-thresholded using Image J program (NIH, USA) to subtract background staining. Relative Tim-3 fluorescence intensity in different brain cells at one day after ICH was measured by the ratio of the fluorescence intensity of each group to the fluorescence intensity of the astrocyte. The fluorescence intensity in each cell area was calculated. ROI were selected within the ipsilateral (IL) around the hematoma.

The number of M1 microglia/macrophages (CD16/32+/CD11b+ cells), or M2 microglia/macrophages (CD206+/CD11b+ cells) at one day after ICH was counted from one microscopic field randomly selected around the hematoma area (within 300μm to the hematoma).

1. **Fluoro-Jade B staining and TUNEL staining**

**5.1 Fluoro-Jade B staining**

Fluoro-Jade B (FJB) staining was performed as described previously.[^4^](#_ENREF_4)^,^ [^5^](#_ENREF_5) The brain sections were incubated with 0.06% KMnO_4_ solution in the dark at room temperature for 15 min. Then the brain sections were washed with PBS (5 min/time) for 3 times, and were incubated with FJB working solution (containing 0.1% acetic acid) for 60 min. Subsequently, the sections were washed 3 times by PBS (5 min/time) and air-dried at room temperature in the dark. Finally, the brain sections were observed under a fluorescence microscope, and Image J software (NIH, USA) was used to analyze the positive rate of FJB.

**5.2 TUNEL staining**

TUNEL staining was performed as described previously.^[6](#_ENREF_6)^ According to the manufacturer’s protocol (Roche, Mannheim, Germany), firstly, staining slides were incubated with the TUNEL reaction mixture for 1 h at 37 °C and with NeuN-neuronal marker (Abcam, UK,) overnight at 4 °C. Then, a secondary antibody (Alexa Fluor 555 Donkey Anti-Mouse IgG, Life Technologies, USA) was added, and the degree of cell death was visualized via a fluorescence microscope (Olympus BX50/BX-FLA/DP70; Japan). The TUNEL-positive cells were counted by an observer who was blind to the experimental groups. To evaluate the extent of cell death, index of cell death was defined as the average number of TUNEL-positive cells/neurons in each section at each rat.

**6.** **Brain Water Content Measurement**

Rats were anesthetized with chloral hydrate (4% chloral hydrate 10 ml / kg, i.p.) and decapitated after ICH 24 h later for brain water content measurement. The Rats’ brains were immediately removed and cut a 4 mm thickness coronal brain slice from the frontal pole 4 mm. The brain slice is divided into four samples: contralateral basal ganglia and cortex, ipsilateral basal ganglia and cortex. In addition, the cerebellum group is as a control. These brain tissue samples were weighed by an electronic analytical balance (Model FA 2004; Yueping Scientific Instrument Co., Ltd, Shanghai, China) to obtain sample wet weight (WW). Brain tissue was placed in an oven at 100 ℃ for 24 hours to obtain the dry weight (DW). Brain tissue water content (%) is calculated as [(WW − DW)/WW] × 100.

**7. Immunoprecipitation analysis**

Immunoprecipitation tests were performed as reported previously [^6^](#_ENREF_6). Firstly, the brain samples were lysed in ice-cold RIPA lysis buffer (BeyoTime Institute of Biotechnology, China). For immunoprecipitation, the lysate was incubated with specific antibodies or rabbit IgG (negative control) overnight at 4°C with agitation. Protein A+G Sepharose beads were then added to each immune complex, and the lysate-bead mixture was incubated for 4h at 4°C with rotary agitation. The beads were washed 3 times with RIPA lysis buffer and then eluted with 4× SDS loading buffer. Western blot assay was then performed for further protein separation and detection.

**8. The relative Tim-3 fluorescent intensity between sham and 1 d group is shown.** Data are mean± SD. ****p* < 0.001 vs. sham group, n=6 (Fig. S4).

**Supplement Figure 4**

**
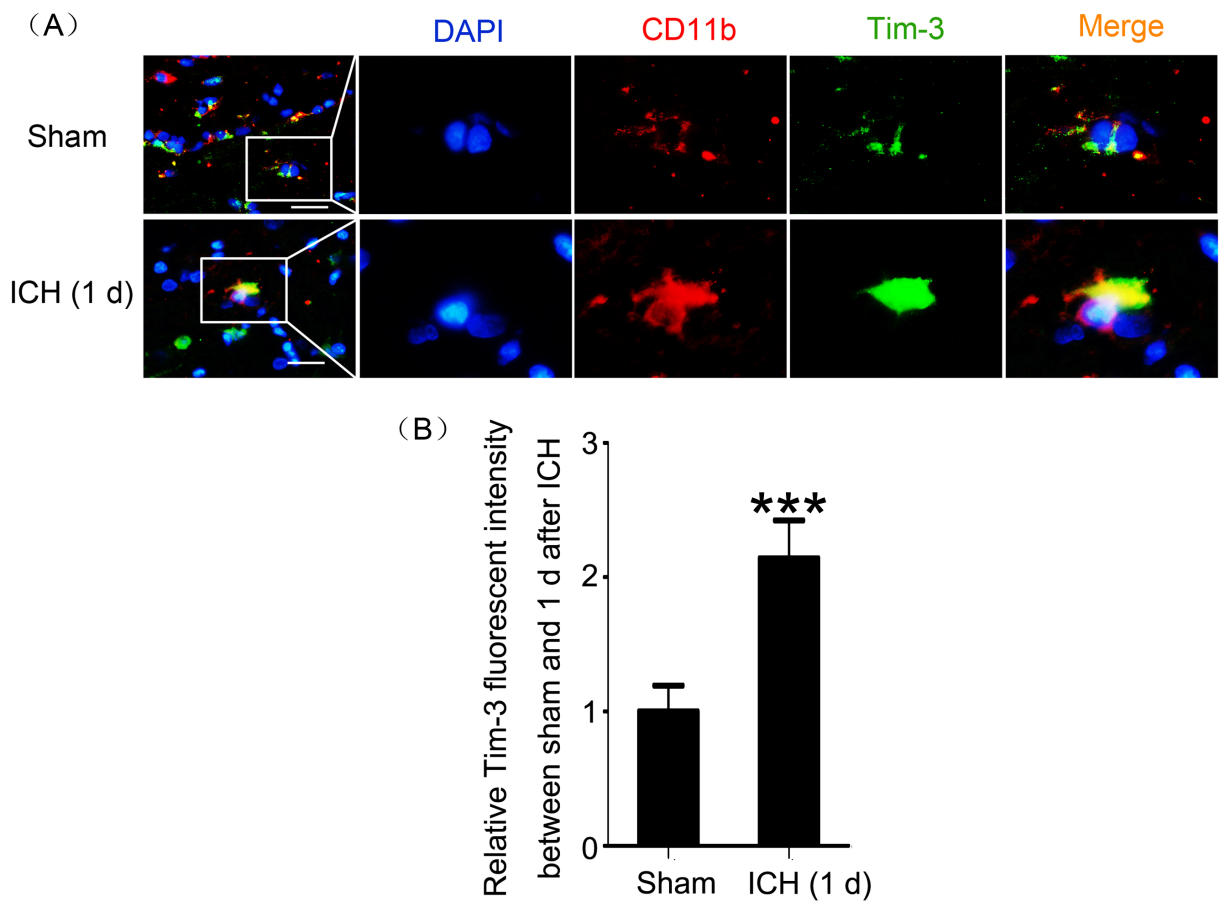
**

**References**

1. de G. The rat hypothalamus in stereotaxic coordinates. *The Journal of comparative neurology*. 1959; 113: 389-400.

2. You W, Wang Z, Li H, et al. Inhibition of mammalian target of rapamycin attenuates early brain injury through modulating microglial polarization after experimental subarachnoid hemorrhage in rats. *Journal of the neurological sciences*. 2016; 367: 224-31.

3. Li H, Gao A, Feng D, et al. Evaluation of the protective potential of brain microvascular endothelial cell autophagy on blood-brain barrier integrity during experimental cerebral ischemia-reperfusion injury. *Translational stroke research*. 2014; 5: 618-26.

4. Zhu HT, Bian C, Yuan JC, et al. Curcumin attenuates acute inflammatory injury by inhibiting the TLR4/MyD88/NF-kappaB signaling pathway in experimental traumatic brain injury. *Journal of neuroinflammation*. 2014; 11: 59.

5. Lin S, Yin Q, Zhong Q, et al. Heme activates TLR4-mediated inflammatory injury via MyD88/TRIF signaling pathway in intracerebral hemorrhage. *Journal of neuroinflammation*. 2012; 9: 46.

6. Shen H, Chen Z, Wang Y, et al. Role of Neurexin-1beta and Neuroligin-1 in Cognitive Dysfunction After Subarachnoid Hemorrhage in Rats. *Stroke*. 2015; 46: 2607-15.
